# Satisfaction with online education among students, faculty, and parents before and after the COVID-19 outbreak: Evidence from a meta-analysis

**DOI:** 10.3389/fpsyg.2023.1128034

**Published:** 2023-02-13

**Authors:** Tianyuan Xu, Ling Xue

**Affiliations:** ^1^Centre for Humanities and Social Sciences, University of Wales Trinity Saint David, Lampeter, United Kingdom; ^2^School of Foreign Languages, Zhongnan University of Economics and Law, Wuhan, China

**Keywords:** online education, student satisfaction, faculty satisfaction, parent satisfaction, COVID-19, meta-analysis

## Abstract

The COVID-19 pandemic has presented a unique opportunity for the advancement of online education, as billions of students from 190 countries have been compelled to take classes remotely. The degree of satisfaction is considered one of the major factors in determining the quality of online educational programs. As a result, many empirical studies have been conducted on the level of satisfaction with online education over the last two decades. However, few studies have synthesized previous findings from similar research questions. Therefore, to reinforce statistical power, the study aimed to conduct a meta-analysis to examine satisfaction with online education among students, faculty, and parents before and after the COVID-19 outbreak. A total of 52 studies in English were screened from six academic electronic databases, yielding 57 effect sizes using Comprehensive Meta-Analysis (CMA) software. The results showed that the prevalence of satisfaction with online education among students, faculty, and parents before and after the COVID-19 outbreak was 59.5, 75.3, and 70.7%, respectively, with a significant difference between the satisfaction rates of students and those of their faculty and parents. Besides, we conducted a moderator analysis that found that (1) a significantly higher number of students in the pre-pandemic era in countries with developed digital infrastructure and emergency online learning environments were less satisfied with online education than their counterparts in the post-pandemic era, in countries with developing digital infrastructure, and in non-emergency online learning environments. Additionally, a significantly higher proportion of adult education learners reported being satisfied with online education compared to K-12 and university students. (2) The faculty in the non-emergency situation reported almost double the satisfaction rate of their counterparts in the emergency circumstance. With fewer satisfied remote learning students, efforts could be made by providing well-designed online lessons *via* faculty and strengthening digital infrastructure *via* governments to improve student satisfaction.

## 1. Introduction

As far back as the 1990s, online education has been researched and applied as a supplement to traditional face-to-face classroom learning (Kang et al., 2022). Since then, the pace of development in online education has accelerated along with the thriving technology industry, and an increasing number of students are engaging in the modern era of the digital world (Prasad, 2022). The spread of the coronavirus disease 2019 (COVID-19) pandemic also gave rise to the online education era. Approximately 1.5 billion students of all ages and levels of education in 190 countries have enrolled in online courses at home to prevent and control COVID-19 (Garcia, 2022; WHO, 2022).

Categorized by temporal constraint, online learning can be roughly divided into two types: asynchronous and synchronous (Persada et al., 2022). Asynchronous online learning refers to the acquiring of knowledge by students from online curricular materials on their own time, and thus, the teacher and students remain independent in both time and space (Friend and Johnston, 2005; Murphy et al., 2010). Despite being less constrained by time, asynchronous online learning indicates a higher demand for students' self-discipline due to limited interaction with instructors (Persada et al., 2022). Unlike asynchronous online learning, another type, synchronous online learning, requires students and teachers to schedule the same time for spontaneously communicating as if they were physically co-present despite being geographically independent (Murphy et al., 2010). Therefore, some scholars believe that asynchronous online learning is “individually based,” whereas synchronous online learning is “more like classroom instruction” (Bernard et al., 2004).

Nevertheless, after the outbreak of the COVID-19 pandemic, a brand-new concept, emergency remote education, was put forward, enriching the category of online education. Emergency remote learning refers to a sudden change of instructional delivery to an alternate mode owing to a grave crisis (Tunc and Toprak, 2022). One of the primary misalignments between emergency remote education and traditional online education is that the faculty under the former circumstance is usually deficient in preparing lessons due to time limitations (Ferri et al., 2020; Fuchs, 2022).

Admittedly, online education holds advantages in many aspects, such as convenience, better interaction, and learning effectiveness, but various disadvantages were also reported, including technical obstacles, poor academic performance, and a lack of practical knowledge (Kotrikadze and Zharkova, 2021; Dinh and Nguyen, 2022). To perceive whether advantages prevail over disadvantages, assessing the degree of satisfaction is regarded as one of the major indicators in determining the quality of online educational programs (Dziuban et al., 2015). The definition of satisfaction with online education is multidimensional, relating to factors such as workload, technological support, feedback, and pedagogical skills (Öztürk et al., 2020; Wei and Chou, 2020). From different perspectives, student and faculty satisfaction can be defined as an attitude consequent upon the evaluation of educational experience, services, and facilities (Weerasinghe and Fernando, 2017), while parent satisfaction is not limited to learning itself but includes extra-learning factors such as children's screen time and physical exercise (Harjule et al., 2021).

Reviewing the literature regarding online education satisfaction has aroused scholars' interest far longer than the duration of the COVID-19 pandemic. Over the past two decades, studies conducted in America found that 83.4% of faculty and 88% of students expressed satisfaction with asynchronous online education (Hartman et al., 2000; Swan, 2001). Regarding synchronous online education, 88.5% of faculty at a university in America and 83% of students at a university in Australia reported that they were satisfied with completely online education in the pre-pandemic era (Palmer and Holt, 2009; Wasilik and Bolliger, 2009). After the COVID-19 outbreak, researchers focused on the satisfaction level in emergency remote education. For instance, 49% of students from 12 universities in Romania and 80.7% of parents of primary school students in China were satisfied or very satisfied with emergency online learning (Rucsanda et al., 2021; Zheng et al., 2022). However, numerous empirical studies have concentrated on satisfaction with online learning from students', faculty's, and parents' perspectives before and after the COVID-19 outbreak. Few studies have integrated previous findings from the same research question. Therefore, to aggregate data with a stronger statistical power than any specific study, the study aimed to conduct a meta-analysis based on empirical studies in English to examine satisfaction with online education before and after the COVID-19 outbreak. To reflect the satisfaction rate from a more comprehensive perspective, the current study explores the research question from threefold standpoints: students, faculty, and parents.

## 2. Method

In this section, methods are introduced to display the integral research process of the current study. Previous studies were first filtered according to the inclusion and exclusion criteria from six academic databases. Then, data were extracted and coded from screened original articles. Statistical analyses, such as investigating heterogeneity, were performed as the last procedure.

### 2.1. Search strategy

The current study systematically searched six academic electronic databases, including Web of Science, Scopus, ProQuest, EBSCO, PubMed, and Google Scholar, before 5 December 2022. To search and include as many related articles as possible, use search terms (i.e., online education, online learning, emergency remote learning, emergency remote teaching, remote education, virtual learning, distance learning, e-learning, student satisfaction, faculty satisfaction, instructor satisfaction, parent satisfaction). All the possible combinations of these keywords were input in the search bar with the following string: (“online education” OR “online learning” OR “emergency remote learning” OR “emergency remote teaching” OR “remote education” OR “virtual learning” OR “distance learning” OR “e-learning”) AND (“student satisfaction” OR “faculty satisfaction” OR “instructor satisfaction” OR “parent satisfaction”).

### 2.2. Inclusion and exclusion criteria

Only studies that met the following filter criteria were deemed eligible and included in this meta-analysis: (1) empirical studies that were available in English (since English is a worldwide lingua franca, scholars from different nations would report achievements in scientific research in English); (2) studies that reported the prevalence of satisfaction with online education; and (3) studies that accurately reported the number of participants. Conversely, studies were excluded if (1) study subjects were not students, faculty, or parents; (2) study subjects were clinical populations (patients with physical or mental disease); (3) studies were not original research but case reports, editorials, reviews, or commentaries; and (4) studies reported duplicate data.

### 2.3. Data extracting and coding

The data from all included studies were extracted. A predetermined table was designed for coding the extracted information with the following variables: author, publication year, country, learning phase, major, asynchronous or synchronous online education, whether or not emergency remote learning, sample size, and satisfaction rate. Tables 1–3 show the summary coding of the included student, faculty, and parent satisfaction studies.

**Table 1:** Summary coding of included student satisfaction studies.

**References**	**Country**	**Learning phase**	**Major**	**Asynchronous or synchronous**	**Whether or not ERL**	**Sample size**	**Rate**
Abdous (2019)	USA	University	/	Synchronous	No	3936	69.40%
Agapito and Japos (2021)	Philippines	University	Engineering	Synchronous	No	168	37.76%
Al-Balas et al. (2020)	Jordan	University	Medical	Synchronous	No	652	26.77%
Aldhahi et al. (2021)	SA	University	Diverse	Synchronous	Yes	1226	51.00%
Al-omairi and Hew (2022)	Malaysia	University	/	Synchronous	No	3649	85.80%
Ansar et al. (2020)	Pakistan	University	Diverse	Synchronous	No	600	22.00%
Arain et al. (2022)	SA	University	Medical	Synchronous	Yes	209	30.00%
Cole et al. (2014)	USA	University	Diverse	Synchronous	No	472	58.70%
Cui et al. (2021)	China	K-12	/	Synchronous	No	867	73.90%
Elshami et al. (2021)	UAE	University	Medical	Synchronous	No	370	41.30%
Fiorini et al. (2022)	Malta	AE	/	Synchronous	No	82	88.90%
Garratt-Reed et al. (2016)	Australia	University	psychology	Synchronous	No	56	83.00%
Holmes et al. (2019)	UK	University	/	Synchronous	No	47784	81.00%
Ke and Xie (2009)	USA	AE	Diverse	Synchronous	No	128	91.10%
Li et al. (2021)	China	University	Medical	Synchronous	No	230	36.50%
Maqableh (2021)	Jordan	University	/	Synchronous	Yes	483	29.40%
Mir et al. (2022)	Pakistan	University	/	Asynchronous	No	732	59.00%
Mohamed et al. (2021)	Egypt	University	Diverse	Synchronous	No	782	49.70%
Naciri et al. (2022)	Morocco	University	Medical	Synchronous	No	330	53.30%
Naseer and Rafique (2021)	Pakistan	University	Diverse	Synchronous	No	406	37.70%
Olson et al. (2005)	USA	AE	Diverse	Synchronous	No	70	94.00%
Palmer and Holt (2009)	Australia	University	/	Synchronous	No	761	44.80%
Pelucio et al. (2022)	Brazil	University	Diverse	Synchronous	No	152	29.60%
Potrč et al. (2020)	Slovenia	K-12	/	Synchronous	No	1844	61.47%
Ristić Dedić and Jokić (2021)	Croatia	K-12	/	Synchronous	No	920	41.20%
Rodrigues et al. (2022)	Portugal	University	Medical	Synchronous	No	415	77.00%
Rucsanda et al. (2021)	Romania	University	Music	Synchronous	Yes	220	49.00%
Ruiz-Grao et al. (2022)	Spain	University	Medical	Synchronous	No	139	58.00%
Swan (2001)	USA	University	Diverse	Asynchronous	No	1406	88.00%
Toprak and Tunc (2022)	Turkey	University	Medical	Synchronous	Yes	2290	66.70%
Wolf and Peyre (2018)	USA	University	Medical	Asynchronous	No	30	95.80%
Yekefallah et al. (2021)	Iran	University	/	Synchronous	No	420	41.00%
Zheng et al. (2022)	China	K-12	/	Synchronous	Yes	781	57.00%

Rate, represents satisfaction rate; /, represents the information was unreported; AE, represents adult education; ERL, represents emergency remote learning.

**Table 2:** Summary coding of included faculty satisfaction studies.

**References**	**Country**	**Learning phase**	**Major**	**Asynchronous or synchronous**	**Whether or not ERL**	**Sample size**	**Rate**
Almeda and Rose (2000)	USA	University	Writing	Synchronous	No	9	66.67%
Alqahtani et al. (2022)	SA	University	Diverse	Synchronous	No	1117	71.00%
Arain et al. (2022)	SA	University	Medical	Synchronous	Yes	13	46.00%
Bedriñana et al. (2022)	Peru	University	Diverse	Synchronous	Yes	1029	25.00%
Benito et al. (2021)	Costa Rica, India and Turkey	University	/	Synchronous	Yes	22	82.60%
Chen et al. (2022)	China	K-12	/	Synchronous	No	13730	69.32%
Elshami et al. (2021)	UAE	University	Medical	Synchronous	No	70	74.30%
Evans and Myrick (2015)	USA	University	/	Asynchronous	No	162	66.00%
Fauzi and Khusuma (2020)	Indonesia	K-12	/	Synchronous	Yes	45	20.00%
Fredericksen et al. (2019)	USA	University	/	Asynchronous	No	105	100%
Hartman et al. (2000)	USA	University	/	Asynchronous	No	30	83.40%
Li et al. (2021)	China	University	Medical	Synchronous	No	95	61.10%
McLawhon and Cutright (2012)	USA	University	/	Synchronous	No	110	95.00%
Saini et al. (2021)	India	University	Medical	Synchronous	No	159	96.90%
Seoane et al. (2021)	Spain	K-12	Writing	Synchronous	No	158	84.00%
Truzoli et al. (2021)	Italy	K-12	/	Synchronous	No	107	62.60%
Vishwanathan et al. (2021)	India	University	Diverse	Synchronous	No	104	92.20%
Wasilik and Bolliger (2009)	USA	University	/	Synchronous	No	102	88.50%

Rate, represents satisfaction rate; /, represents the information was unreported; ERL, represents emergency remote learning.

**Table 3:** Summary coding of included parent satisfaction studies.

**References**	**Country**	**Learning phase**	**Major**	**Asynchronous or synchronous**	**Whether or not ERL**	**Sample size**	**Rate**
Butz (2003)	USA	K-12	/	Synchronous	No	186	86.02%
Cui et al. (2021)	China	K-12	/	Synchronous	No	867	77.90%
Joseph et al. (2021)	India	K-12	/	Synchronous	No	300	20.00%
Lau et al. (2021)	China	K-12	/	Synchronous	No	3381	53.10%
Rathaliya et al. (2022)	India	K-12	/	Synchronous	No	220	89.00%
Zheng et al. (2022)	China	K-12	/	Synchronous	Yes	781	80.70%

Rate, represents satisfaction rate; /, represents the information was unreported; ERL, represents emergency remote learning.

### 2.4. Statistical analysis

The results of the current meta-analysis were analyzed by the Comprehensive Software Meta-Analysis (CMA), which is one of the most commonly used software packages to conduct a meta-analysis due to its extensive analytic options and simple interface (Brüggemann and Rajguru, 2022). To calculate the overall satisfaction rate, the software first converted input ratio data into logit data using the formula *logit* = *Log*(*p*/(1−*p*)) and then transformed logit data back into ratio data *via* the formula $var\left(logit\right)\;=\;\frac{1}{case}+\frac{1}{non-case}$ (Card, 2012). Then, two methods were used to examine moderating effects related to various variables. For continuous variables, meta-regression was used, whereas a subgroup analysis was conducted for categorical variables. In the subgroup analysis, the number of effect sizes under the same moderating variable should be no less than three to guarantee the representativeness of the studies under that certain variable (Zhang et al., 2021).

Heterogeneity was investigated to decide which statistical model (the fixed-effect model or the random-effect model) should be applied. Measuring heterogeneity by I^2^ is a crucial evaluation criterion, with an I^2^ > 75% regarded as a cutoff point for choosing the random-effect model, whereas the fixed-effect model should be applied (Huedo-Medina et al., 2006). Besides, Egger's regression test was widely utilized for measuring whether publication bias exists in the meta-analysis because it is more precise and sensitive (Egger et al., 1997; Lin et al., 2018). A sensitivity analysis was also performed to evaluate the robustness of the results.

## 3. Results

The study results are presented in the following subsections.:First, search results and sample characteristics are described, followed by assessments of heterogeneity and publication bias. Then, we will present the combined effect, sensitivity, and moderator analysis for satisfaction among students, faculty, and parents.

### 3.1. Search results and sample characteristics

**Figure 1:**
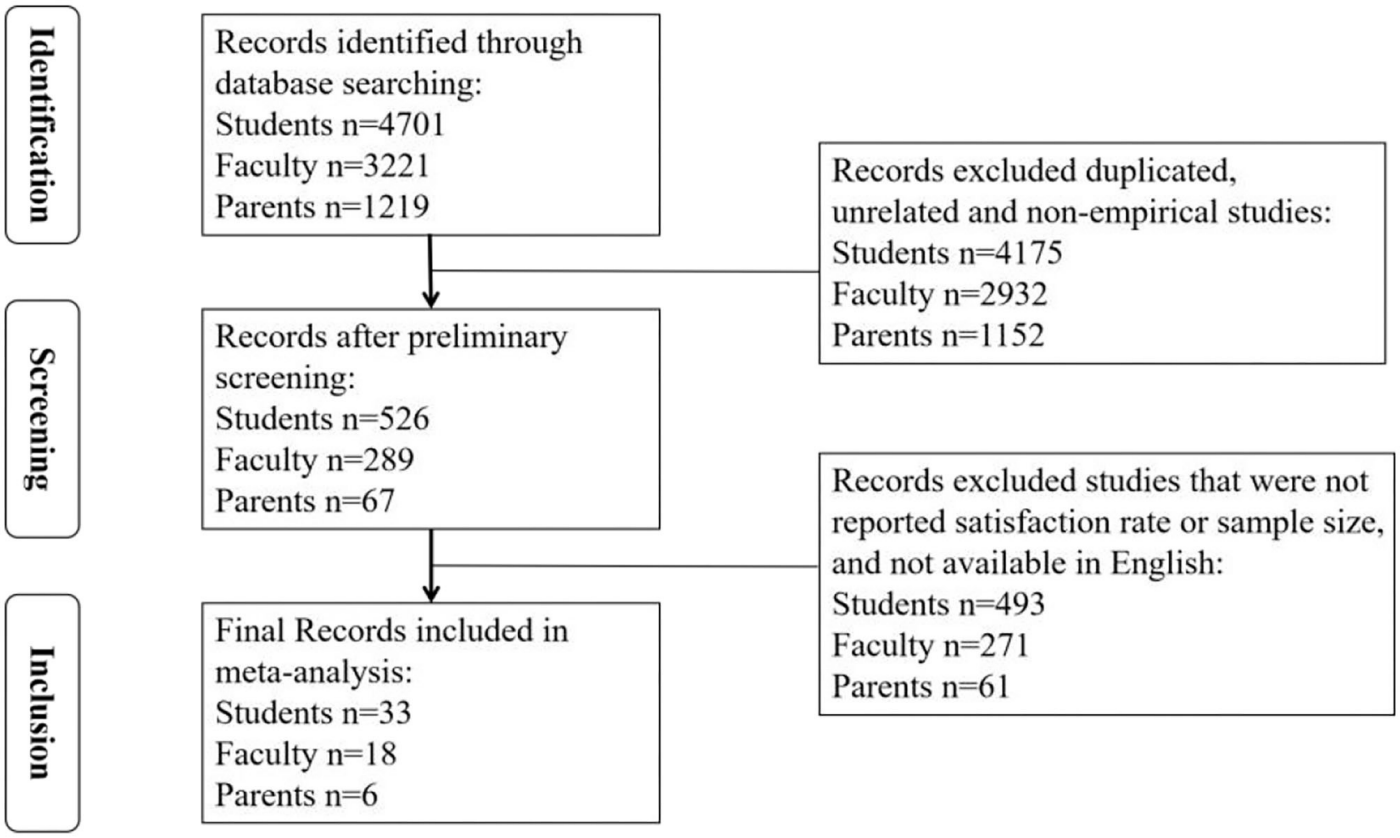
A flow diagram of Literature Searching and Screening.

The study screening process is illustrated in Figure 1. Studies on online education satisfaction among students, faculty, and parents were initially identified in six academic electronic databases. After screening based on the abovementioned inclusion and exclusion criteria, 57 effect sizes (33 for students; 18 for faculty; 6 for parents) were generated from 52 research studies (28 reported only student satisfaction; 15 reported only faculty satisfaction; 4 reported only parent satisfaction; 3 reported both student and faculty satisfaction; and 2 reported both student and parent satisfaction).

Overall, a total of 93,686 participants of different ages were included in this meta-analysis. Included studies were conducted in 26 countries (i.e., Australia, Brazil, China, Costa Rica, Croatia, Egypt, India, Indonesia, Iran, Italy, Jordan, Malaysia, Malta, Morocco, Pakistan, Peru, the Philippines, Portugal, Romania, Saudi Arabia, Slovenia, Spain, Turkey, the United Arab Emirates, the United Kingdom, and the United States), covering both developed and developing countries from six continents, namely Africa, Asia, Europe, North America, Oceania, and South America.

### 3.2. Investigating heterogeneity and publication bias

**Figure 2:**
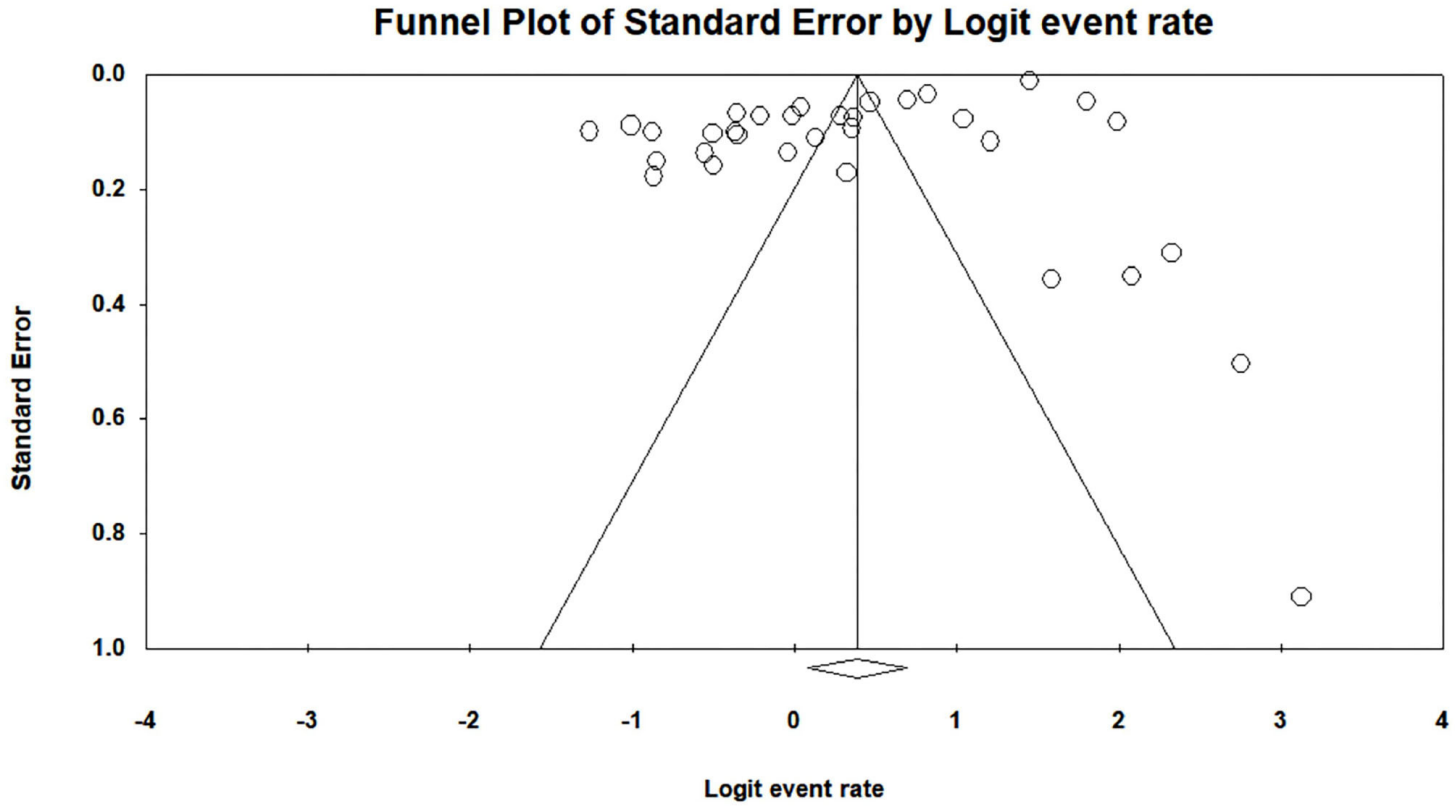
A funnel plot assessing publication bias of satisfaction with online education among students before and after the COVID-19 outbreak.

**Figure 3:**
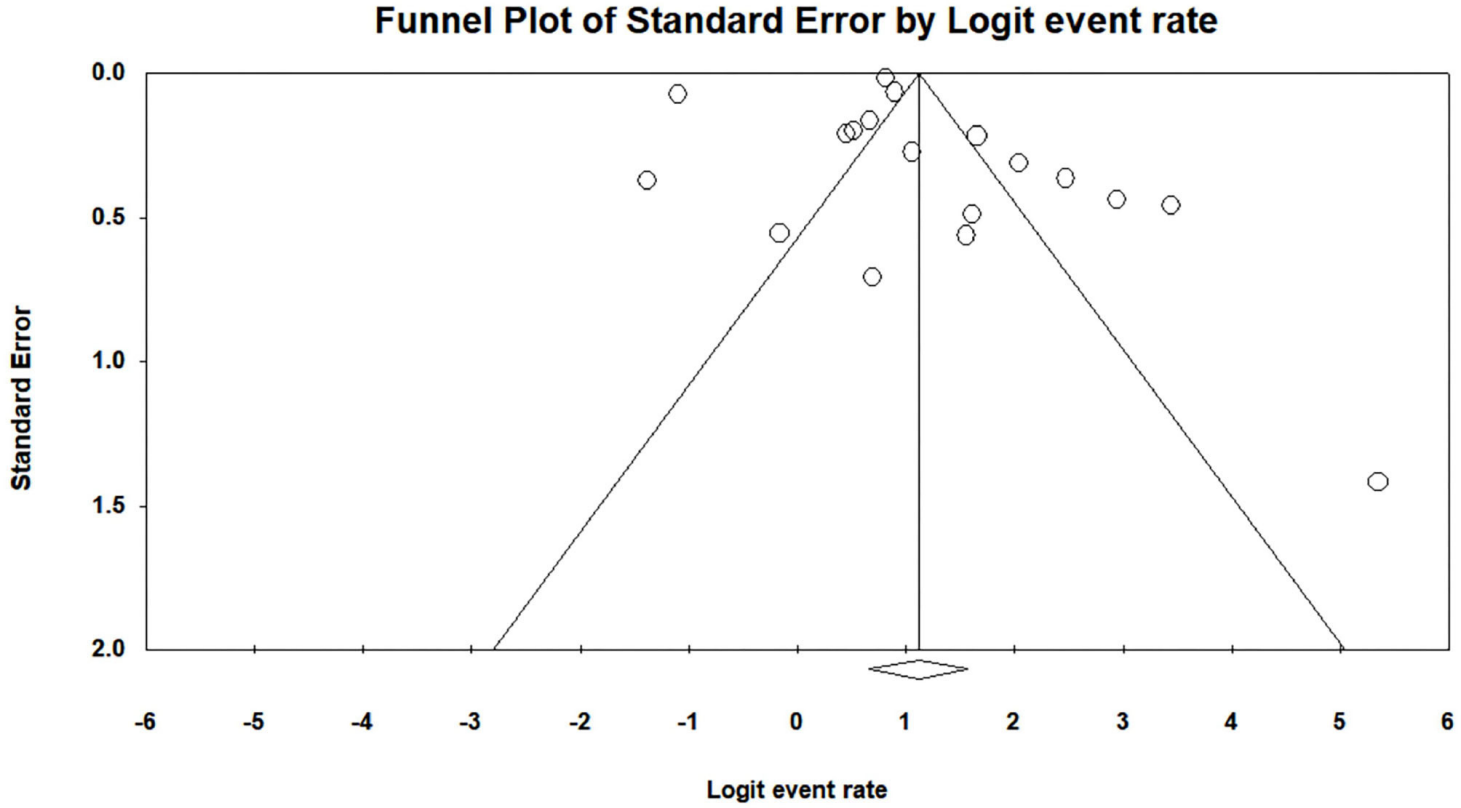
A funnel plot assessing publication bias of satisfaction with online education among faculty before and after the COVID-19 outbreak.

**Figure 4:**
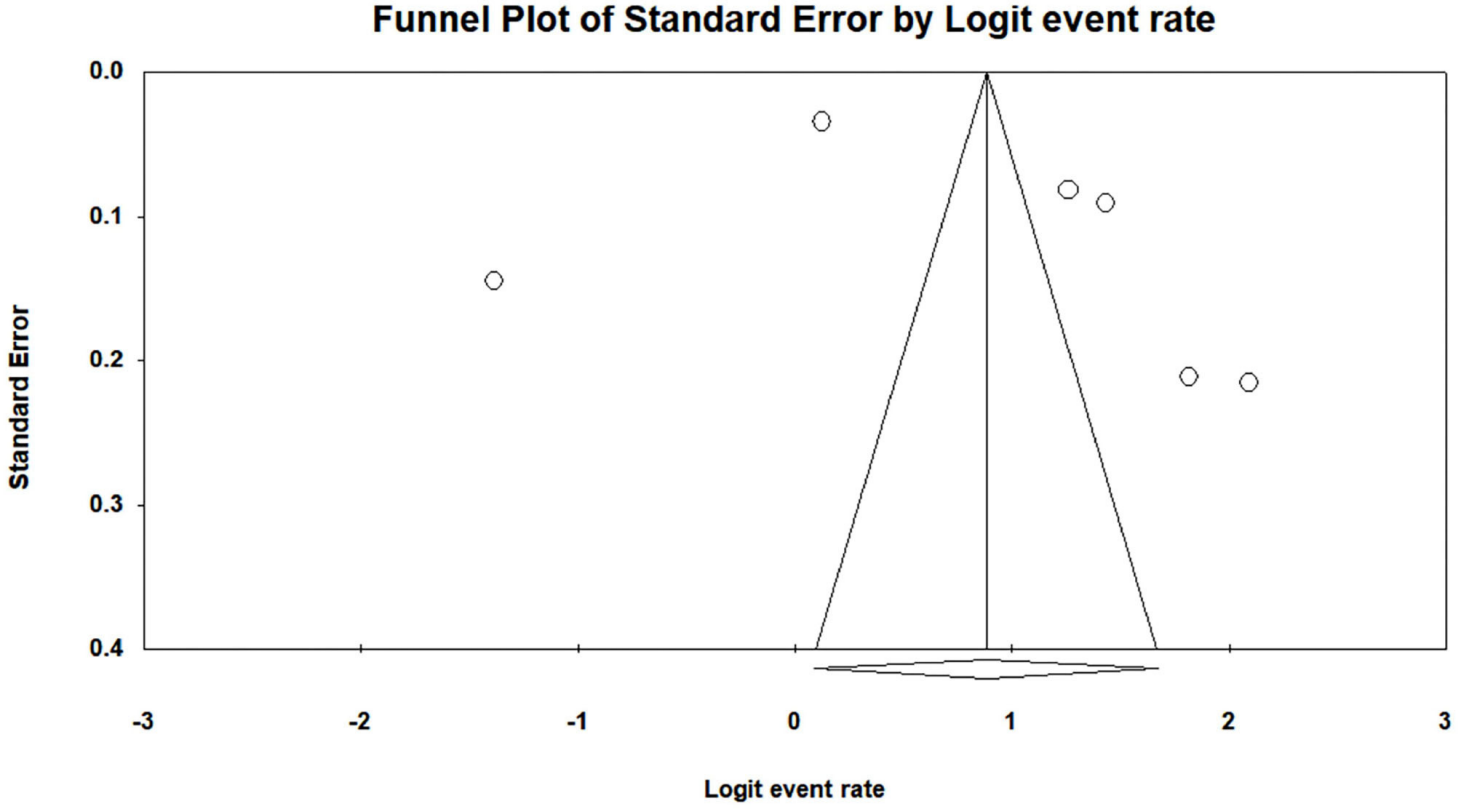
A funnel plot assessing publication bias of satisfaction with online education among parents before and after the COVID-19 outbreak.

Heterogeneity test results are displayed in Table 4. It shows that all I^2^ results were >75%, and the Q test was significant in student (*P* = 0.000, *I*^2^ = 99.48), faculty (*P* = 0.000, *I*^2^ = 97.99), and parent (*P* = 0.000, *I*^2^ = 99.11) satisfaction studies. Thus, the current study selected the random-effects model as the statistical model (Huedo-Medina et al., 2006). Additionally, the result of Egger's regression test is also shown in Table 4 to assess whether publication bias exists in the meta-analysis. The *P*-value was found to be significant in the studies among students (*p* = 0.00) but not significant in the studies among faculty (*p* = 0.88) or parents (*p* = 0.36), indicating that there was a publication bias that needed to be corrected for studies among students. The trim-and-fill method (Duval and Tweedie, 2000), one of the most commonly used methods, was applied to correct the publication bias found in the studies among the students (Shi and Lin, 2019). A trim-and-fill analysis was performed using STATA statistical software with the “metatrim” command (Alimoradi et al., 2022). The “metatrim” result showed that “no trimming was performed, and the data remained unchanged,” indicating that the current student results were robust. The funnel plot assessing publication bias in satisfaction with online education among students, faculty, and parents before and after the COVID-19 outbreak is shown in Figures 2–4.

**Table 4:** Heterogeneity and publication bias test.

**Satisfaction**	**Heterogeneity**		**Egger's**
	***P***	***I** ^2^*	***P***
Student	0.00	99.48	0.00
Faculty	0.00	97.99	0.88
Parent	0.00	99.11	0.36

### 3.3. Meta-analysis overall results

Based on the random-effects model, the overall satisfaction rate toward online education among students, faculty, and parents before and after the COVID-19 outbreak was 65.3% (95% CI = [0.603, 0.700]). Furthermore, a sensitivity analysis was carried out, which showed that the prevalence of satisfaction fluctuated between 64.7 and 66.1% after a random individual study was removed from the overall data. The little difference between the results before and after the sensitivity analysis indicates that the results are highly reliable.

The subgroup analysis by roles is displayed in Table 5, which shows that, ranging from low to high, 59.5% of students (95% CI = [0.519, 0.666]), 70.7% of parents (95% CI = [0.522, 0.842]), and 75.3% of faculty (95% CI = [0.660, 0.827]) were satisfied with online education before and after the COVID-19 outbreak. In addition, the moderating effect of role (*p* = 0.026) shows that students were much less happy with online learning than teachers and parents. The following sections will show the specific satisfaction results from the threefold perspective of students, faculty, and parents.

**Table 5:** Satisfaction with online education among students, faculty, and parents before and after the COVID-19 outbreak and role moderator analysis.

**Role**	**k**	**Effect size and 95% CI**			**Heterogeneity**	
		**R**	**Lower limit**	**Upper limit**		
Student	33	0.595	0.519	0.666	*Q*	7.293
Faculty	18	0.753	0.660	0.827	*Df*	2
Parent	6	0.707	0.522	0.842	*P*	0.026

### 3.4. Meta-analysis results among students, faculty, and parents

In the current meta-analysis, we analyzed a total of 33 effect sizes and found that 59.5% of students were satisfied with online education. We also examined several variables that could potentially affect satisfaction levels, including the publication year, the timing of the study (before or after the COVID-19 outbreak), whether the online learning was in an emergency, the type of online education (asynchronous or synchronous), the phase of learning (e.g., K-12 or university), and whether the study was conducted in a developed or developing country. The results of this analysis are as follows (see Table 6): (1) The publication year had a significant effect on student satisfaction (b = −0.102, 95% CI = [-0.161,−0.042], *p* < 0.001), indicating that student satisfaction with online education has decreased over the past two decades. (2) The timing of the study (pre- or post-pandemic), whether or not the online learning was in an emergency, the phase of learning (e.g., K-12 or university), and the country's level of development all had significant effects on student satisfaction. Specifically, a significantly higher number of students in the pre-pandemic era (79.7%), emergency online learning situations (62.3%), and developed countries (72.8%) expressed satisfaction with online education compared to their counterparts in the post-pandemic era (50.7%), non-emergency online learning situations (47.1%), and developing countries (45.8%). Additionally, a significantly higher number of adult education learners (91.0%) were satisfied with online studies compared to K-12 (58.9%) or university students (54.8%). (3) The type of online education (synchronous or asynchronous) and students' majors did not affect satisfaction levels significantly.

**Table 6:** Moderator analysis for student satisfaction.

**Moderator variable**	**Heterogeneity**			**Type**	**K**	**r**	**95% CI**	
	***Q***	***df***	***P***				**Lower limit**	**Upper limit**
Before or after the COVID-19 outbreak	21.345	1	0.000	Before	9	0.797	0.712	0.861
				After	24	0.507	0.425	0.589
Whether or not EOL	4.288	1	0.038	Yes	6	0.471	0.357	0.588
				No	27	0.623	0.539	0.700
Asynchronous or synchronous	3.411	1	0.065	Asynchronous	3	0.837	0.559	0.954
				Synchronous	30	0.571	0.490	0.648
Learning phase	64.010	2	0.000	K-12	4	0.589	0.461	0.705
				University	26	0.548	0.459	0.634
				AE	3	0.910	0.870	0.939
Major	0.601	1	0.438	Diverse	9	0.617	0.448	0.761
				Medical	9	0.531	0.392	0.666
Developed or developing country	14.997	1	0.000	Developed	16	0.728	0.646	0.797
				Developing	17	0.458	0.353	0.567

ERL, represents emergency remote learning; AE, represents adult education.

Regarding faculty satisfaction (see Table 7), 75.3% of faculty expressed satisfaction with online education based on 18 effect sizes. We also examined several variables that could potentially affect satisfaction levels among faculty members, including the publication year, whether the teaching was in an emergency online environment, the timing of the study (before or after the COVID-19 outbreak), the type of online education (asynchronous or synchronous), the phase of teaching (e.g., K-12 or university), and whether the study was conducted in a developed or developing country. The results of this analysis are as follows: (1) The publication year did not have a significant effect on faculty satisfaction (b = −0.066, 95% CI = [−0.130, −0.001], *p* = 0.046), indicating that the faculty satisfaction rate hardly changed over time. (2) The effect of whether the teaching was in an emergency online environment was significant (*p* = 0.001); faculty members teaching in a non-emergency situation (79.5%) reported almost double the satisfaction rate of their counterparts in emergencies (40.6%). (3) Other variables, such as the timing of the study (pre- or post-pandemic), the type of online education, the phase of teaching, and the country's level of development, did not significantly affect satisfaction levels.

**Table 7:** Moderator analysis for faculty satisfaction.

**Moderator variable**	**Heterogeneity**			**Type**	**K**	**r**	**95% CI**	
	***Q***	***df***	***P***				**Lower limit**	**Upper limit**
Before or after the COVID-19 outbreak	3.607	1	0.058	Before	6	0.870	0.716	0.947
				After	12	0.694	0.569	0.796
Whether or not EOL	10.840	1	0.001	Yes	4	0.406	0.201	0.650
				No	14	0.795	0.748	0.835
Asynchronous or synchronous	1.061	1	0.303	Asynchronous	3	0.868	0.583	0.969
				Synchronous	15	0.737	0.630	0.821
Teaching phase	3.208	1	0.073	K-12	4	0.618	0.444	0.766
				University	14	0.804	0.663	0.895
Major	0.207	1	0.649	Diverse	3	0.673	0.283	0.915
				Medical	4	0.767	0.501	0.916
Developed or developing country	3.359	1	0.067	Developed	8	0.831	0.721	0.904
				Developing	10	0.682	0.537	0.799

ERL, represents emergency remote learning.

For the parent satisfaction group in this meta-analysis (see Table 8), the number of effect sizes was 6, which is far less than the number for student and faculty satisfaction. Based on the included studies, 70.7% of parents reported satisfaction with online education. However, due to the limited number of studies on parents of students in different education levels (except for K-12), on remote learning in emergencies, on other majors, and in developed countries, we could not conduct a moderator analysis with these variables. The moderating effect of publication year was non-significant (b = −0.053, 95% CI = [−0.170, −0.064], *p* = 0.375), which suggests that there has been little change in the level of satisfaction among parents over time.

**Table 8:** Moderator analysis for parent satisfaction.

**Moderator variable**	**Heterogeneity**			**Type**	**k**	**r**	**95% CI**	
	***Q***	***df***	***P***				**Lower limit**	**Upper limit**
Before or after the COVID-19 outbreak	/	/	/	Before	/	/	/	/
				After	/	/	/	/
Whether or not EOL	/	/	/	Yes	/	/	/	/
				No	/	/	/	/
Asynchronous or synchronous	/	/	/	Asynchronous	/	/	/	/
Synchronous					/	/	/	/
Phase	/	/	/	K-12	/	/	/	/
				University	/	/	/	/
Major	/	/	/	Diverse	/	/	/	/
				Medical	/	/	/	/
Developed or developing country	/	/	/	Developed	/	/	/	/
				Developing	/	/	/	/

ERL, represents emergency remote learning; /, represents the number of studies is insufficient to report related data.

## 4. Discussion

In light of the aforementioned results, a discussion will revolve around the feasible explanation of these findings, integrating them with relevant prior research. In addition, limitations are discussed to guide follow-up studies in the future.

### 4.1. Main effect analysis

The current study is the first meta-analysis examining satisfaction with online education among students, faculty, and parents before and after the COVID-19 outbreak in English. It synthesized the reported data from previous empirical studies from a threefold perspective over the past two decades. After applying strict inclusion and exclusion criteria, the study included data from 52 studies with 93,686 participants from 26 countries, resulting in 57 effect sizes (33 for students, 18 for faculty, and 6 for parents). Overall, the results of this meta-analysis showed that 59.5% of students, 75.3% of faculty, and 70.7% of parents were happy with online education before and after the COVID-19 outbreak. There was a big difference between the satisfaction rates of students and those of their faculty and parents.

The current findings are broadly consistent with the few previous analogous meta-analysis findings. Some scholars concluded that 63.8% of overall students were satisfied with e-learning after the outbreak of the COVID-19 pandemic (Nakhoda et al., 2021), a slight difference from the result of 59.5% in this study. The results suggest that around 60% of students find online learning an effective method for acquiring knowledge and staying on track with their coursework. There is limited research on meta-analyses of faculty and parent satisfaction with online education.

### 4.2. Moderating effect analysis

The study used moderator analysis to examine the effect of several variables, including the publication year, the timing of the study (before or after the COVID-19 outbreak), whether the study was conducted during an emergency online learning period, the type of online education (asynchronous or synchronous), the phase of learning (K-12, university, or adult education), the students' major, and whether the study was conducted in a developed or developing country. Regarding the publication year, the moderating effect was only significant among students (fewer students reported satisfaction with distance education over the past two decades). In contrast, faculty and parent satisfaction were unaffected over time. The different evaluations of satisfaction can explain the reason behind this discrepancy. From the perspective of students, their satisfaction is associated with the value of the learning experience since they tend to construct a knowledge system in the social context of interacting with faculty and other students, engaging in activities, and receiving feedback (Bandura, 2001; Thurmond et al., 2002; Elshami et al., 2021). Three major categories influence student satisfaction with e-learning: faculty, interactivity, and technology (Bolliger, 2004; Kurucay and Inan, 2017). Unlike students, faculty satisfaction is defined as the perception of efficiency, effectiveness, and benefit during the online teaching process. Students, instructors, and institutions are three key factors determining faculty satisfaction with online education (Bolliger and Wasilik, 2009; Bolliger et al., 2014). From parents' perspective, factors affecting their satisfaction are not limited to learning outcomes but also extra-learning elements such as children's screen time, exposure to harmful website content, and lack of time for physical exercise (Harjule et al., 2021). It indicates that only students' satisfaction is highly susceptible to interactivity in online education. After the outbreak of COVID-19, students were compelled to engage in isolated distance learning at home. Thus, they cannot interact with classmates or teachers as actively as before the pandemic, which diminishes their satisfaction with online study. Nonetheless, faculty and parent satisfaction are not strongly associated with interactivity and are thus unaffected by limiting interactions with other social members.

The second moderator analysis of “before or after the COVID-19 outbreak” also showed that only the number of students expressing satisfaction with online education significantly declined, but the number of faculty remained stable. Apart from lower interactivity following the pandemic, there is a significant difference in mental health between students and faculty following the COVID-19 outbreak. The prevalence of anxiety, depression, and stress was high among students in the online learning context after the pandemic. For instance, 70.7, 64, and 48.3% of pharmacy students reported mild-to-severe anxiety, depression, and stress levels in Lebanon (Hammoudi Halat et al., 2022) and 51.3% (anxiety), 29.4% (depression), and 56.5% (stress) of university students in Malaysia (Moy and Ng, 2021). However, regarding the prevalence of mental disorders among lecturers, only 15.7, 17.6, and 21.6% of them showed anxious, depressive, and/or stress-related symptoms (Miguel et al., 2021), which was much less than that of students. According to studies that found that anxious people are less satisfied with doctor consultations, mental health problems could negatively influence related satisfaction (Tanis et al., 2016). Therefore, a significantly smaller number of students than faculty were satisfied with online education following the COVID-19 outbreak.

The variable “whether or not emergency online learning” showed that satisfaction in the context of emergency online learning was significantly different from that in the non-emergency circumstance among both students and faculty. As many as 62.3% of students and 79.5% of faculty in the non-emergency situation reported satisfaction, the rate decreased to 47.1 and 40.6%, respectively, when switching to an emergency pattern. Unlike traditional online education, emergency online learning refers to a temporary shift to an alternate delivery mode due to crisis circumstances (Ferri et al., 2020; Hodges et al., 2020; Fuchs, 2022). Due to the sudden transition, faculty usually prepare and design lessons insufficiently and lack professional training in technological support systems (O'Keefe et al., 2020). Therefore, emergency online education could passively impact many dimensions, including user satisfaction, academic performance, and mental health (Lu et al., 2003; El-Sakran et al., 2022). In addition, since student and faculty satisfaction are interrelated (Yildiz, 2018), it is reasonable to conclude that there is a significant difference in satisfaction with online education between emergency and non-emergency situations among both students and faculty.

Regarding the following variable, asynchronous or synchronous online education, the moderating effect of it was nonsignificant both for students and faculty. The current result is consistent with previous meta-analysis findings that showed satisfaction was higher, but negligibly so in a synchronous environment such as webinars than in asynchronous online instruction (Ebner and Gegenfurtner, 2019). It indicates that asynchronous or synchronous online education is not influencing learning or teacher satisfaction. Unlike emergency online education, educators can have adequate time to prepare lessons in an asynchronous or synchronous context, thus not affecting the satisfaction rate. Many scholars pointed out that asynchronous recording can be regarded as a necessary alternative and additional tool for students who cannot attend synchronous lessons (Bixler et al., 2021; Manou et al., 2022).

The learning phase is a moderator variable that also shows significant results among students rather than faculty. The present results found that a significantly higher number of adult education learners (91.0%) were satisfied with online study than K-12 (58.9%) or university students (54.8%). The term “adult learner” is defined as an individual above the age of 24 who is employed full-time, studies part-time, and usually needs to support dependents such as a spouse and parents at home (Forbus et al., 2011; Ng and Baharom, 2018). Hence, unlike full-time K-12 or university students, adult learners are swamped with balancing commitments such as job, family, and education (Bishop, 2002). The flexibility of online education exactly meets the need to pursue an academic degree while balancing career and family commitments for adult students (Alexander et al., 2009). For this reason, most adults are willing to participate in online educational programs and are highly motivated and task-oriented (Merriam and Caffarella, 1991; Cercone, 2008). Therefore, compared with K-12 or university students who attend online courses passively, more adult learners are satisfied with online study.

For majors, no significant difference was shown between students and faculty in medical and other diverse majors in satisfaction with online education before and after the COVID-19 outbreak. Many scholars believe that implementing online education in majors highly dependent on firsthand experiences, such as medical science, is much more challenging than other subjects (Patra et al., 2021; Nikas et al., 2022). Nevertheless, medical students and faculty satisfaction were not reported to be different from their counterparts in other majors, which is inseparable from technological support. Owing to the development of digital technology, operations and clinical skills traditionally learned and acquired in laboratories and hospitals are currently feasible in online education (Li et al., 2021), and most faculty (77%) regard virtual teaching applications as a convenient tool (Arain et al., 2022). Hence, students and faculty in medical science were roughly as satisfied as others who specialized in other majors with online education.

The last moderator variable in this meta-analysis is developed or developing countries, which shows that a significantly higher number of students in developed countries (72.8%) were satisfied with online education than their counterparts in developing countries (45.8%), but there was no significant difference between faculty in developed and developing countries. The reason for it also lies behind the different determining factors affecting students' and faculty's satisfaction with online education: faculty, interactivity, and technology for students (Bolliger, 2004; Kurucay and Inan, 2017), whereas students, instructors, and institutions for faculty (Bolliger and Wasilik, 2009; Bolliger et al., 2014). For developing countries, the quality and quantity of e-content and e-resources are far from enough, which is caused by a lack of digital infrastructure (Adnan and Anwar, 2020). Students in rural and underprivileged areas are particularly affected by technological issues such as poor Internet connections and the incompatibility of digital learning platforms with their electronic devices (Adedoyin and Soykan, 2020; Zarei and Mohammadi, 2021). Since student satisfaction is more likely to be influenced by technological issues, it is understandable that more students, not faculty, reported satisfaction with distance education than their counterparts in developing countries.

### 4.3. Limitations and future study

Despite being a pioneering study examining satisfaction with online education among students, faculty, and parents before and after the COVID-19 outbreak, this meta-analysis has several limitations. First, the number of studies and moderator variables on parent satisfaction is insufficient, which leads to the moderating effect of variables such as the COVID-19 outbreak, whether or not online learning is in an emergency, and the children's learning phase being unable to be examined. Therefore, more studies related to parents' satisfaction can be collected and further analyzed in the future. Second, there are only two categories for major student variables, namely medical and diverse, since the number of studies on other major variables was less than three. The satisfaction of students in other majors, such as arts, literature, psychology, and business, should also be paid attention to by scholars, as distinct features characterize different disciplines. Third, this study only included published papers and papers written in English. Although publication bias in the meta-analysis is sometimes unavoidable, including unpublished papers and papers in other languages could reduce bias to some extent. Therefore, future follow-up studies could be conducted to explore satisfaction with online education based on more unpublished research and papers in other languages.

## 5. Conclusion

In this meta-analysis, by aggregating evidence from 52 studies conducted over time with 93,686 participants across 26 countries, it was found that the prevalence of satisfaction with online education among students, faculty, and parents before and after the COVID-19 outbreak was 59.5, 75.3, and 70.7%, respectively, with a significant difference between satisfaction rates of students and those of their faculty and parents. Regarding students, a significantly higher number of students in the pre-pandemic era, non-emergency online environment, and developed countries were more satisfied with online education than their counterparts in the post-pandemic era, emergency online environment and developing countries. Moreover, a significantly higher number of adult education learners were satisfied with online study than K-12 and university students. In terms of faculty, instructors in the non-emergency situation reported almost double the satisfaction rate of their counterparts in emergency circumstances. Therefore, student satisfaction was the lowest compared with faculty and parent satisfaction, and measures can be implemented from four perspectives. First, faculty should put more effort into preparing and designing online courses, which will benefit students and themselves. Second, students should be more encouraged to engage in virtual educational activities. Engaged, responsive, and motivated students tend to express satisfaction, which further contributes to an efficient academic atmosphere (Dziuban et al., 2015). Third, governments, especially in developing countries, should enhance the digital infrastructure to provide more stable internet connections and richer e-resources for students attending online courses. Fourth, e-learning digital platforms should emphasize making their platforms compatible with electronic devices used in developing countries. The result of this meta-analysis is expected to contribute to the field of online education. Educators who specialize in online education hope to understand satisfaction with online education from three standpoints and thus could improve student, faculty, and parent satisfaction constructively.

## Data availability statement

The original contributions presented in the study are included in the article/supplementary material, further inquiries can be directed to the corresponding author.

## Author contributions

TX is responsible for conceptualization, software, and writing original draft. LX is responsible for supervision, reviewing, and editing. All authors contributed to the article and approved the submitted version.
